# Comparison of Rubber Band Ligation and Hemorrhoidectomy in Patients With Symptomatic Hemorrhoids Grade III: A Multicenter, Open-Label, Randomized Controlled Noninferiority Trial

**DOI:** 10.1097/DCR.0000000000003679

**Published:** 2025-02-14

**Authors:** Justin Y. van Oostendorp, Lisette Dekker, Susan van Dieren, Ruben Veldkamp, Willem A. Bemelman, Ingrid J.M. Han-Geurts

**Affiliations:** 1 Department of Surgery, Proctos Kliniek, Bilthoven, The Netherlands; 2 Department of Surgery, Amsterdam UMC, Location AMC, Amsterdam, The Netherlands; surgeon at the Proctos Kliniek; surgeon at the Amsterdam University Medical Center (UMC); surgeon at Groene Hart Ziekenhuis; surgeon at Maastricht UMC; surgeon at Onze Lieve Vrouwe Gasthuis; surgeon at Central Military Hospital Utrecht; surgeon at Ijsselland ziekenhuis; surgeon at Diakonessenhuis; surgeon at Flevoziekenhuis; surgeon at Proctos Kliniek; epidemiologist/statistician at Department of Surgery Amsterdam UMC

**Keywords:** Goligher, Grade III, Hemorrhoidectomy, Hemorrhoids, Quality of life, Rubber band ligation

## Abstract

**BACKGROUND:**

The optimal management strategy for grade III hemorrhoids remains a subject of ongoing debate. Hemorrhoidectomy is the criterion standard, but rubber band ligation offers a less invasive outpatient alternative. Treatment variability persists due to a lack of consensus on the preferred strategy.

**OBJECTIVE:**

To directly compare the effectiveness of rubber band ligation and hemorrhoidectomy in the treatment of grade III hemorrhoids.

**DESIGN:**

Open-label, parallel-group, randomized controlled noninferiority trial.

**SETTINGS:**

Multicenter study across 10 Dutch hospitals from October 2019 to September 2022.

**PATIENTS:**

Patients (aged 18 years or older) with symptomatic grade III (Goligher) hemorrhoids were included in this study. Exclusion criteria included prior rectal/anal surgery, more than 1 rubber band ligation/injection within the preceding 3 years, rectal radiation, preexisting sphincter injury, IBD, medical unfitness for surgery (ASA higher than 3), pregnancy, or hypercoagulability disorders.

**INTERVENTIONS:**

Randomized 1:1 to rubber band ligation or hemorrhoidectomy, with up to 2 banding sessions allowed.

**MAIN OUTCOME MEASURES:**

Primary outcomes included 12-month health-related quality of life and recurrence rate. Secondary outcomes included complications, pain, work resumption, and patient-reported outcome measures.

**RESULTS:**

Eighty-seven patients were randomly assigned (47 rubber band ligation vs 40 hemorrhoidectomy). Rubber band ligation was not noninferior to hemorrhoidectomy in quality-adjusted life years (–0.045; 95% CI, –0.087 to –0.004). Recurrence rate was worse in the rubber band ligation group (47.5% vs 6.1%), with an absolute risk difference of 41% (95% CI, 24%–59%). Complication rates were comparable. Pain scores after hemorrhoidectomy were higher during the first week (visual analogue scale 4 vs 1; *p* = 0.002). Rubber band ligation group returned to work sooner (1 vs 9 days; *p* = 0.021). Patient-reported hemorrhoidal symptom scores favored hemorrhoidectomy.

**LIMITATIONS:**

The primary limitation of the study was its early termination due to funding constraints, resulting in a relatively small sample size and limited statistical power. Patient recruitment was hindered by significant treatment preferences and the COVID-19 pandemic.

**CONCLUSIONS:**

Hemorrhoidectomy may benefit patients with grade III hemorrhoids in terms of quality of life, recurrence risk, and symptom burden, whereas rubber band ligation allows faster recovery with less pain. These findings can guide clinical decision-making. See **Video Abstract**.

**CLINICAL TRIAL REGISTRATION NUMBER:**

NCT04621695.

**COMPARACIÓN DE LA LIGADURA CON BANDA ELÁSTICA Y LA HEMORROIDECTOMÍA EN PACIENTES CON HEMORROIDES SINTOMÁTICAS GRADO III: ENSAYO MULTICÉNTRICO, ABIERTO, ALEATORIZADO, CONTROLADO Y DE NO INFERIORIDAD:**

**ANTECEDENTES:**

La estrategia óptima de tratamiento para las hemorroides grado III sigue siendo un tema de debate continuo. La hemorroidectomía es el estándar de oro, pero la ligadura con banda elástica ofrece una alternativa ambulatoria menos invasiva. La variabilidad del tratamiento persiste debido a la falta de consenso sobre la estrategia preferida.

**OBJETIVO:**

Comparar directamente la eficacia de la ligadura con banda elástica y la hemorroidectomía en el tratamiento de las hemorroides grado III.

**DISEÑO:**

Ensayo de no inferioridad, controlado, aleatorizado, de grupos paralelos y abierto.

**ENTORNO CLINICO:**

Estudio multicéntrico en 10 hospitales holandeses desde octubre de 2019 hasta septiembre de 2022.

**PACIENTES:**

Pacientes (≥18 años) con hemorroides sintomáticas grado III (Goligher). Exclusiones: cirugía rectal/anal previa, >1 ligadura con banda elástica/inyección en los tres años anteriores, radiación rectal, lesión preexistente del esfínter, enfermedad inflamatoria intestinal, incapacidad médica para la cirugía (ASA >3), embarazo o trastornos de hipercoagulabilidad.

**INTERVENCIONES:**

Aleatorización 1:1 para ligadura con banda elástica o hemorroidectomía, con hasta dos sesiones de ligadura permitidas.

**PRINCIPALES MEDIDAS DE RESULTADOS:**

Primarias: calidad de vida relacionada con la salud a los 12 meses y tasa de recurrencia. Secundarias: complicaciones, dolor, reanudación del trabajo y medidas de resultados informadas por el paciente.

**RESULTADOS:**

Se aleatorizaron ochenta y siete pacientes (47 ligadura con banda elástica frente a 40 hemorroidectomía). La ligadura con banda elástica no fue no inferior a la hemorroidectomía en años de vida ajustados por calidad (-0,045, intervalo de confianza del 95 %: -0,087 a -0,004). La tasa de recurrencia fue peor en el grupo de ligadura con banda elástica (47,5 % frente a 6,1 %), con una diferencia de riesgo absoluto del 41 % (intervalo de confianza del 95 %: 24 %–59 %). Las tasas de complicaciones fueron comparables. Las puntuaciones de dolor posthemorroidectomía fueron más altas durante la primera semana (escala analógica visual 4 frente a 1; *p* = 0,002). El grupo de ligadura con banda elástica volvió al trabajo antes (1 frente a 9 días; *p* = 0,021). Las puntuaciones de síntomas hemorroidales notificadas por los pacientes favorecieron a la hemorroidectomía.

**LIMITACIONES:**

La principal limitación del estudio fue su finalización temprana debido a las limitaciones de financiación, lo que dio lugar a un tamaño de muestra relativamente pequeño y a un poder estadístico limitado. El reclutamiento de pacientes se vio obstaculizado por las preferencias significativas de tratamiento y la pandemia de COVID-19.

**CONCLUSIONES:**

La hemorroidectomía puede beneficiar a los pacientes con hemorroides de grado III en términos de calidad de vida, riesgo de recurrencia y carga de síntomas, mientras que la ligadura con banda elástica permite una recuperación más rápida con menos dolor. Estos hallazgos pueden orientar la toma de decisiones clínicas. *(Traducción— Dr. Francisco M. Abarca-Rendon*)

**NÚMERO DE REGISTRO DEL ENSAYO CLÍNICO:**

NCT04621695.

Hemorrhoidal disease is a common anorectal condition characterized by symptoms such as bleeding, pain, itching, soiling, and prolapse, which can significantly affect quality of life (QoL).^1^ In the Netherlands, its prevalence is 13.4 per 1000 patients.^2^ The Goligher classification system categorizes hemorrhoids by the degree of hemorrhoidal prolapse and guides treatment strategies.^3^

Grade III hemorrhoids represent a particularly complex management scenario due to inconclusive guidelines regarding optimal treatment strategy.^4,5^ Although hemorrhoidectomy is traditionally considered the criterion standard, rubber band ligation (RBL) offers a simpler, less invasive outpatient alternative with fewer complications and lower patient burden.^6,7^ However, RBL has a recurrence rate of up to 50%, potentially requiring repeat procedures.^8^ Although hemorrhoidectomy generally has lower recurrence rates, it is associated with more postoperative discomfort and a longer recovery period.^7^

A consensus on the best treatment strategy for grade III hemorrhoids is lacking, leading to variability in clinical practice.^9–11^ In 2016, 2 large randomized controlled trials (RCTs) compared stapled hemorrhoidopexy with hemorrhoidectomy and hemorrhoidal artery ligation with RBL.^6,8^ However, a direct comparison of RBL with hemorrhoidectomy is still missing. The present study aimed to address this gap by evaluating the clinical outcomes and cost-effectiveness of RBL versus hemorrhoidectomy in grade III hemorrhoids, focusing on health-related quality of life (HR-QoL) and recurrence rates, and incorporating patient-reported outcomes measures (PROMs) to determine patient preferences.

## MATERIALS AND METHODS

This study adhered to the consolidated standards of reporting trials guidelines.^12^ The HollAND study protocol was published in 2021.^13^ Protocol amendments made after trial commencement are provided in see Supplemental Table 1 at http://links.lww.com/DCR/C470.

### Study Design and Participants

This multicenter, open-label, parallel-group, randomized controlled noninferiority trial was conducted across 10 hospitals in the Netherlands. Adults aged 18 years or older with symptomatic grade III hemorrhoids were enrolled.^3^ Exclusion criteria included prior rectal or anal surgery, more than 1 RBL or injection procedure within the preceding 3 years, rectal radiation, preexisting sphincter injury, IBD, medical unfitness for surgery or completion of the trial (ASA higher than 3), pregnancy, or hypercoagulability disorders. Eligible patients were screened, and those who provided written informed consent were randomly assigned. Data collection was performed through a web-based electronic data capture system (Castor EDC). The study was approved by the Medical Ethical Committee Amsterdam UMC on August 1, 2019 (reference MEC number 2019_093) and registered (NCT04621695).

### Important Changes and Premature Termination of the Trial

Recruitment began on October 8, 2019, with eligibility criteria modified on August 20, 2020, to include patients using oral anticoagulants. The trial was prematurely terminated on September 22, 2022, due to slow participant enrollment. Recruitment was hindered by significant preexisting patient treatment preferences (75% of patients favored RBL treatment, whereas 25% opted for hemorrhoidectomy) and was further delayed by the COVID-19 pandemic.

### Randomization and Masking

Participants were randomly assigned (1:1) to RBL or hemorrhoidectomy using a web-based system (Castor EDC), with stratification by center using permuted blocks of random sizes (2, 4, and 6). Neither the recruiters nor the trial project group were able to access the randomization sequence. The study was open label, with no blinding of participants, clinicians, or researchers.

### Interventions

Before randomization, all patients received conservative treatments, including dietary counseling, stool hygiene, toilet habits, fiber supplementation, and local therapies such as vasoconstrictors, prescribed at the surgeon’s discretion per the pragmatic study design.

#### Rubber band ligation

The details of the RBL procedure are outlined in the study protocol.^13^ In brief, up to 2 RBL sessions were permitted if symptoms persisted after the initial procedure, allowing flexibility in addressing ongoing symptoms, consistent with standard clinical practice. Given that RBL treatment often entails repeated sessions for grade III hemorrhoids, this study hypothesized that repeated RBL (up to 2 sessions) would be noninferior to hemorrhoidectomy regarding HR-QoL and recurrence rates while being more cost-effective.

#### Hemorrhoidectomy

Hemorrhoidectomy was performed using either the open technique^14^ or the closed technique,^15^ both of which are aimed at excising hemorrhoidal cushions using electrocautery. A more detailed description of both techniques can be found in the published study protocol.^13^

### Data Collection

Clinical data were collected at baseline, intervention, and 6-week follow-up, with some assessments conducted by phone due to COVID-19. Data included demographics, medical history, Rome IV criteria for chronic constipation, pelvic floor physiotherapy, symptom duration, and previous treatments. Procedural data covered intervention details and complications. The postoperative period covered the first 90 days after treatment, recording complications such as bleeding requiring hospitalization or transfusion, urinary retention, wound discharge, anal fissure, anal fistula, emergency surgery, anal stenosis, fecal incontinence, or death. Data on discharge, readmissions, and further treatments were also collected.

Subsequent data collection through questionnaires occurred at baseline, 1 day, 1 week, 6 weeks, 6 months, 12 months, and 24 months posttreatment. Assessments included the European Quality of Life 5 Dimensions 5 Level (EQ-5D-5L) score,^16^ self-reported recurrence, pain (visual analog scale [VAS]), analgesic use, return to work, and various PROMs: PROM–hemorrhoidal impact and satisfaction score (PROM-HISS),^17^ Hemorrhoid Severity Score (HSS),^18,19^ Vaizey incontinence score,^20^ and proctoPROM.^21^ These PROMs provided comprehensive evaluations of patient outcomes and treatment effectiveness.^22^

### Outcomes

#### Primary outcomes

The primary outcomes included 12-month HR-QoL and recurrence rate:

- HR-QoL: the study evaluated the difference in quality-adjusted life years between the randomized groups, derived from the HR-QoL scores measured during a 12-month posttreatment period. HR-QoL was assessed using the EQ-5D-5L questionnaire, utilizing Dutch wording and population norms (range, –0.446 to 1.0, with 1.0 indicating optimal health).^23^ The primary analysis period was adjusted to 12 months due to early termination.
- Recurrence rate: recurrence was determined by self-reported symptoms and subsequent procedures within 12 months, excluding repeat RBL sessions. Self-reported symptoms were assessed using a dichotomous question following established methods.^7,8^

#### Secondary outcomes

Secondary outcomes included symptom severity (PROM-HISS score 0–5; HSS score 0–15; proctoPROM score 0–50, with 0 being optimum); pain intensity (VAS score 0–10); analgesic use; time to resume work; complications; incontinence (Vaizey score 0-24, with 0 being optimum), persistent symptoms at 6 weeks; need for further treatment; and health-state utility score (EQ-5D-5L). The timing of various assessments is outlined in Supplemental Table 2 at http://links.lww.com/DCR/C470.

### Sample Size

The sample size calculation was based on the noninferiority design for HR-QoL. It was hypothesized that both treatment groups would exhibit equal quality-adjusted life years. With a 1-sided alpha value of 0.025, power of 80%, SD of 0.15, and noninferiority limit of 0.05, a total of 142 patients were required per group. To accommodate for stratification by center, this number was increased to 162 patients per group. The surgical procedure was standardized; therefore, a low variability between centers was expected and an intraclass correlation coefficient of 0.01 was estimated. The total sample size was 360 patients to adjust for a 10% loss to follow-up. However, 87 patients (24%) were enrolled because of early trial termination.

### Statistical Analysis

Statistical analyses were performed using SPSS Statistics 28 (IBM, Armonk, NY). Descriptive methods assessed data quality and homogeneity. Continuous data were presented as mean or median values and categorical data were presented as percentage proportions. A 2-sided *p* value of <0.05 was considered statistically significant.

Primary analyses were conducted in both the intention-to-treat and as-treated populations. The intention-to-treat population included all randomly assigned patients who underwent the intervention and were followed for at least 12 months, excluding those who did not receive any treatment or were lost to follow-up. The as-treated population comprised patients analyzed on the basis of the actual treatment received, including those who crossed over from RBL to hemorrhoidectomy or vice versa. Missing HR-QoL data from the 8 questionnaires over the 2-year period were imputed using predictive mean matching.

For the primary end points, analyses were conducted with a 1-sided alpha value of 0.025. Noninferiority of RBL compared to hemorrhoidectomy was assessed on the basis of QALY difference and recurrence rate. Linear regression with the center as a fixed effect was used to determine noninferiority for HR-QoL, defined as the lower bound of the 95% CI beginning above the –0.05 QALY difference in both the intention-to-treat and as-treated analysis. For recurrence rate, the noninferiority at 12 months postprocedure was assessed against a predefined boundary of a 10% increase of recurrence in the RBL group. Additional comparisons included specific recurrence metrics: further treatments within 12 months and self-reported recurrence up to 24 months.

Secondary outcomes were analyzed using 2-sided *t* tests or Mann-Whitney *U* tests for continuous data and relative risk ratios with 95% CIs for binary data, applying a 2-sided alpha value of 0.05. A random intercept linear regression model analyzed repeated measures from various questionnaires, including received treatment and center as fixed effects. For secondary outcomes, no additional imputation techniques were applied, so only the available data are presented.

### Amendments to the Protocol After Commencement of the Trial

All amendments have been approved by the accredited Medical Ethical Committee Amsterdam UMC (MEC nr 2019_093).

### Preregistration

The present study was preregistered at the Dutch Trial Registry (NTR8020) and at ClinicalTrials.gov (NCT04621695) without a formal statistical analysis plan. In 2021, the study protocol was published with open access.^13^

### Ethical Approval

The study was approved by the Medical Ethical Committee Amsterdam University Medical Center (MEC No. 2019_093).

### Informed Consent

Each patient gave their written informed consent before enrollment in the study and before randomization or data collection.

### Data Availability

Study data are available upon reasonable request.

## RESULTS

### Participant Flow and Recruitment

**FIGURE 1.**
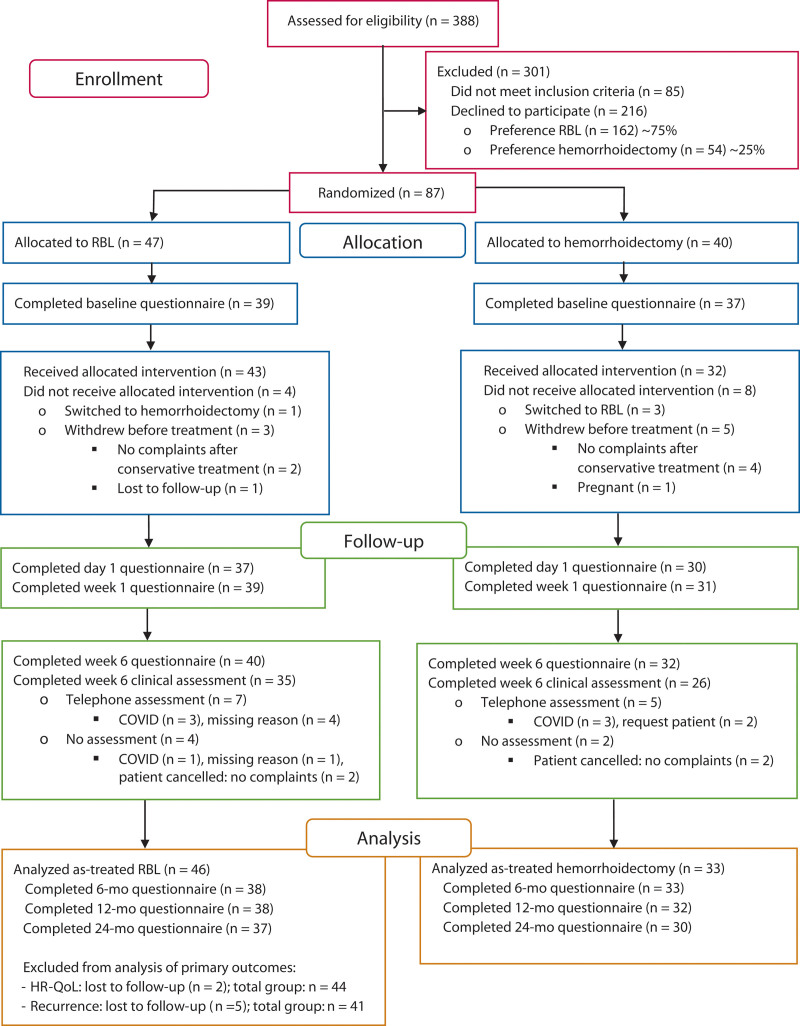
CONSORT flow diagram. CONSORT = consolidated standards of reporting trials; RBL = rubber band ligation.

A consolidated standards of reporting trials flow diagram illustrates the participant flow through the study (Fig. 1). Between October 8, 2019, and September 22, 2022, 388 patients were screened. Of these, 216 declined participation due to treatment preference (75% RBL:25% hemorrhoidectomy). Eighty-seven patients consented and were randomly assigned to RBL (n = 47) or hemorrhoidectomy (n = 40). Six withdrew pretreatment after symptom resolution with conservative measures, 1 patient was excluded because of pregnancy, and 1 patient was lost to follow-up. Thus, 79 patients (91%) received study treatment. Four patients switched groups: 1 from RBL to hemorrhoidectomy, and 3 from hemorrhoidectomy to RBL. Ultimately, 46 patients underwent RBL and 33 patients underwent hemorrhoidectomy (defined as the study population). Twelve-month follow-up was completed by 77 participants (97%), with self-reported recurrence available for 73 participants (92%). At 24 months, 67 participants (85%) completed the questionnaires. Given the consistency of findings between the intention-to-treat and as-treated populations, we report exclusively on the study population.

### Baseline Data

Baseline characteristics, including symptoms and scores, were comparable between the groups (Table 1). Procedural data by treatment group are shown in Table 2. In the RBL group, 9 patients (20%) received same-day treatment postrandomization, compared to none in the hemorrhoidectomy group. The median time from randomization to intervention was 11 days (interquartile range [IQR], 5–30) for RBL and 50 days (IQR, 27–88) for hemorrhoidectomy (*p* < 0.001). Treatments were primarily performed by surgeons. Hemorrhoidectomy was performed under general anesthesia in 94% of cases. In the RBL group, the median number of rubber bands placed was 3 (IQR, 3–4). Additional 1 to 2 cushion hemorrhoidopexy was performed in 15% of cases in the hemorrhoidectomy group.

**TABLE 1.** Baseline characteristics of study participants by treatment group

*Demographics and clinical characteristics*	*RBL* *(n = 46*)	*Hemorrhoidectomy (n = 33*)	*p*
Sex, n (%)			0.809
Male	30 (65)	23 (70)	
Female	16 (35)	10 (30)	
Age, y, mean (SD)	53 (13.0)	51 (12.5)	0.630
BMI, mean (SD)	26.0 (3.8)	25.8 (3.9)	0.882
ASA classification, n (%)			0.118
I	20 (43)	22 (67)	
II	23 (50)	9 (27)	
III	3 (7)	2 (6)	
Smoking, n (%)	7 (15)	7 (21)	0.766
Medical history, n (%)			
Irritable bowel syndrome	1 (2)	2 (6)	0.568
Obstipation	1 (2)	1 (3)	>0.99
Diabetes mellitus	4 (9)	0 (0)	0.136
Rome IV criteria FCC, n (%)	16 (35)	17 (52)	0.240
Previous pelvic floor physiotherapy, n (%)	3 (7)	7 (21)	0.093
Duration of symptoms, mo, median (IQR)	24 (5–78)	36 (8–102)	0.457
Previous RBL treatment, n (%)	18 (39)	15 (46)	0.647
Symptoms (PROM) at baseline, n (%)			
Bleeding	16 (35)	17 (53)	0.343
Pain	22 (48)	16 (49)	0.813
Prolapse	37 (80)	29 (88)	>0.99
Itching	16 (35)	8 (24)	0.215
Soiling	18 (39)	12 (36)	0.632
Symptom scores at baseline^a^			
PROM-HISS score, mean (SD)	2.7 (0.7)	2.5 (0.7)	0.493
HSS score, mean (SD)	7.4 (2.7)	7.9 (3.3)	0.482
ProctoPROM score, mean (SD)	19.9 (10.9)	21.9 (10.8)	0.436
Vaizey score, median (IQR)	4 (0.25–6)	4 (0.25–8)	0.827

FCC = functional chronic constipation; HSS = hemorrhoidal symptom severity; IQR = interquartile range; proctoPROM = proctology patient-reported outcome measure; PROM = patient reported outcome measure; PROM-HISS = PROM–Hemorrhoidal Impact and Satisfaction Score; RBL = rubber band ligation.

a Retrieved from baseline questionnaires (RBL, n = 40; hemorrhoidectomy, n = 36).

**TABLE 2.** Procedural clinical data by treatment group

*Clinical characteristics*	*RBL (N = 46*)	*Hemorrhoidectomy (N = 33*)	*p*
Treatment performed on the same day as randomization, n (%)	9 (20)	0 (0)	0.009
Time to procedure, d, median (IQR)	11 (5–30)	50 (27–88)	<0.001
Day cases, n (%)	46 (100)	31 (94)	0.171
Treatment performed by surgical grade, n (%)			0.568
Surgeon	45 (98)	31 (94)	
Surgical resident	1 (2)	2 (6)	
Type of anesthesia, n (%)			NA
General	NA	31 (94)	
Spinal	NA	2 (6)	
Amount of rubber bands placed, median (IQR)	3 (3–4)	NA	NA
Surgical technique, n (%)			NA
Open (Milligan-Morgan)	NA	8 (24)	
Closed (Ferguson)	NA	23 (70)	
Other: Ligasure	NA	2 (6)	
No. of cushions excised, n (%)			NA
1 cushion	NA	15 (46)	
2 cushions	NA	12 (36)	
3 cushions	NA	6 (18)	
Additional treatment during the procedure, n (%)	0 (0)	7 (21)	NA
Skin tag excision	0 (0)	2 (6)	
Hemorrhoidopexy (1 or 2 cushions)	NA	5 (15)	
Postoperative complications, n (%)	3 (6)	4 (12)	0.443
Postoperative bleeding requiring reintervention	0 (0)	1 (3)	
Urinary retention requiring catheterization	1 (2)	0 (0)	
Wound discharge	0 (0)	2 (6)	
Anal fissure	2 (4)	1 (3)	
Anal stenosis	0 (0)	0 (0)	
Fecal incontinence (Vaizey score)	0 (0)	0 (0)	
Medication at discharge, n (%)			
Paracetamol	24 (51)	32 (97)	<0.001
NSAIDs	6 (13)	30 (91)	<0.001
Opioids	1 (2)	17 (52)	<0.001
Clinical assessment after 6 wk, n (%)			0.908
Outpatient clinic	35 (76)	26 (79)	
Telephone	7 (15)	5 (15)	
No assessment	4 (9)	2 (6)	
Proctoscopy performed at 6 wk, n (%)	17 (37)	6 (18)	0.067
Hemorrhoidal tissue still present	16 (35)	0 (0)	<0.001
Additional outpatient procedures, n (%)			
Excision of residual skin tags	6 (11)	1 (3)	0.392

IQR = interquartile range; NA = not applicable; NSAID = nonsteroidal anti-inflammatory drug; RBL = rubber band ligation.

At 6 weeks, physical clinical assessments were completed for 76% of RBL and 79% of hemorrhoidectomy participants. Telephone assessments were conducted for 15% of RBL and 13% of hemorrhoidectomy participants due to COVID-19 or other reasons. Clinical assessments were not conducted for 6 participants, as detailed in Figure 1.

### Health-Related Quality of Life

**FIGURE 2.**
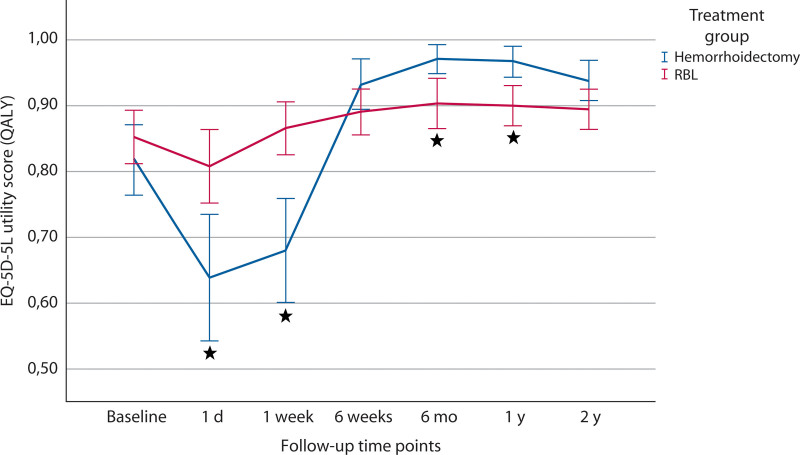
Health-related quality-of-life scores during 2-y follow-up. EQ-5D-5L = European Quality of Life 5 Dimensions 5 Level; QALY = quality-adjusted life year; RBL = rubber band ligation. *p < 0.05. Brackets indicating 95% confidence intervals.

Over 12 months, the mean QALY score was 0.898 (SD 0.10) for the RBL group and 0.942 (SD 0.07) for the hemorrhoidectomy group (Table 3). Linear regression showed a significant difference favoring hemorrhoidectomy (mean difference –0.045; 95% CI, –0.087 to –0.004; *p* = 0.016), with the lower bound of the 95% CI exceeding the noninferiority limit. Figure 2 illustrates HR-QoL trends from baseline to 2 years. Although baseline scores were comparable (*p* = 0.259), the RBL group initially exhibited higher scores up to week 1. From 6 weeks onward, hemorrhoidectomy group scores were higher, with both groups leveling off by 2 years.

**TABLE 3.** HR-QoL during 12 mo in QALYs

*QALY 12 mo*	*RBL (N = 44*)	*Hemorrhoidectomy (N = 33*)	*Mean difference* *(95% CI*)	*p* ^a^
Mean (SD)	0.898 (0.10)	0.942 (0.07)	–0.045 (–0.087 to –0.004)	0.016
Median (IQR)	0.917 (0.85–0.97)	0.967 (0.91–0.99)		

Linear regression analysis, corrected for center.

IQR = interquartile range; HR-QoL = health-related quality of life; QALY = quality-adjusted life year; RBL = rubber band ligation.

a One-sided alpha.

### Recurrence Rate

At 12 months, recurrence was observed in 48.8% of RBL participants versus 6.1% in the hemorrhoidectomy group, with an absolute risk difference (ARD) of 43% (95% CI, 25%–60%; *p* < 0.001), exceeding the noninferiority limit of 10% (Table 4). Self-reported recurrence was higher in the RBL group (24%) compared to none in the hemorrhoidectomy group (ARD 24%; 95% CI, 10%–37%; *p* = 0.003). In addition, 26% of RBL participants required more than 1 additional treatment, compared to 3% in the hemorrhoidectomy group (ARD 23%; 95% CI, 9%–37%; *p* = 0.006). Specifically, 3 RBL participants (6.5%) underwent multiple RBL sessions (3×, 3×, and 6×), 3 participants (6.5%) underwent 1 extra RBL session followed by hemorrhoidectomy, and 6 participants (13%) underwent immediate hemorrhoidectomy after initial RBL failure. Thirteen RBL participants (28%) underwent 1 repeated RBL session, as was allowed by the study protocol (see Supplemental Table 3 at http://links.lww.com/DCR/C470).

**TABLE 4.** Recurrence rate at 12 mo

*Recurrence rate*	*RBL*	*Hemorrhoidectomy*	*ARD (95% CI*)	*p* ^a^
Total recurrence rate,^b^ n (%)	20/41 (48.8)	2/33 (6.1)	0.43 (0.25 to 0.60)	<0.001
Self-reported recurrence, n (%)				
6 wk	6/23^c^ (26.1)	1/22^c^ (4.5)	0.22 (0.02 to 0.42)	0.096
6 mo	3/38^c^ (7.9)	1/33^c^ (3)	0.05 (–0.05 to 0.15)	0.618
12 mo	9/38^c^ (23.7)	0/32^c^ (0)	0.24 (0.10 to 0.37)	0.003
Further treatment, n (%)	12/46 (26.1)	1/33 (3)	0.23 (0.09 to 0.37)	0.006
≥2 RBL	3 (6.5)	–		
1 RBL + hemorrhoidectomy	3 (6.5)	–		
Immediate hemorrhoidectomy	6 (13)	1 (3)		

ARD = absolute risk difference; RBL = rubber band ligation.

a Fisher exact test and 2-sided *p* values.

b Some participants had both self-reported recurrences and underwent further treatment.

c Denominator is the number of patients returning the questionnaire.

At 6 weeks, 26% of RBL participants and 4.5% of hemorrhoidectomy participants reported unchanged or worsened symptoms (ARD 22%; 95% CI, 2%–42%). At 6 months, these rates were 7.9% versus 3% (ARD 5%; 95% CI, –5% to +15%). At 24 months, self-reported recurrence was 29.7% in the RBL group versus 6.7% in the hemorrhoidectomy group (ARD 23%; 95% CI, 6%–40%; see Supplemental Table 4 at http://links.lww.com/DCR/C470).

### Complications

Postoperative complications were observed in 6% of patients in the RBL group and 12% of patients in the hemorrhoidectomy group (Table 2), with similar rates of serious adverse events. Incontinence scores improved by about 1 unit in both groups, with no significant differences during follow-up (see Supplemental Table 5 at http://links.lww.com/DCR/C470).

### Pain and Use of Analgesics

Postoperative pain scores were similar on day 1 (median VAS 4 vs 6, *p* = 0.071) but higher in the hemorrhoidectomy group by week 1 (median VAS 1 vs 4, *p* = 0.002), consistent with their worst pain experience (median VAS 3 vs 8, *p* < 0.001; Table 5). More hemorrhoidectomy participants used analgesics at day 1 (97% vs 73%) and week 1 (100% vs 48%), although most used paracetamol or nonsteroidal anti-inflammatory drugs, with 33% still using opioids. Analgesic use lasted shorter in the RBL group (median 3 vs 7 days, *p* < 0.001). By week 6, pain scores and analgesic use were similar between the groups (Table 6).

**TABLE 5.** Postoperative pain scores

*Postoperative pain scores*	*RBL*	*Hemorrhoidectomy*	*p* ^a^
Average pain (VAS), median (IQR)			
Day 1	4 (1–7)	6 (3–7.5)	0.071
Week 1	1 (0–5)	4 (2.75–6.25)	0.002
Week 6	0 (0–1)	0 (0)	0.279
Worst pain (VAS), median (IQR)			
Week 1	3 (1–8)	8 (5.75-9)	<0.001
Week 6	1 (0–3)	0 (0–1)	0.031

The VAS ranged from 0 to 10, with 0 meaning zero pain and 10 meaning worst pain imaginable. IQR = interquartile range; RBL = rubber band ligation; VAS = visual analog scale.

a Mann-Whitney *U* test.

**TABLE 6.** Postoperative use of analgesics

*Use of analgesics*	*RBL*	*Hemorrhoidectomy*	*RR (95% CI*)
Day 1, n (%)	27 (73)^a^	28 (97)^a^	0.76 (0.61–0.93)
Week 1: number of days, median (IQR)	3 (2–6)	7 (6.75–7)	*p* < 0.001^b^
Paracetamol, n (%)	19 (48)^a^		0.44 (0.32–0.62)
NSAIDs, n (%)	10 (23)^a^		0.32 (0.17–0.61)
Opioids, n (%)	1 (2)^a^		0.07 (0.01–0.55)
Week 6			
Paracetamol, n (%)	2 (9)^a^	1 (4)^a^	1.91 (0.24–15.5)

IQR = interquartile range; NSAID, nonsteroidal anti-inflammatory drug; RBL = rubber band ligation; RR = relative risk ratio.

a Denominator is the number of patients returning the questionnaire.

b Mann-Whitney *U* test.

### Resumption of Work

Eighty-five percent of RBL participants returned to work within the first week, compared to 44% in the hemorrhoidectomy group (RR 2.0; 95% CI, 1.76–2.25), with a significantly shorter median return-to-work time (1 vs 9 days, *p* = 0.021; Table 7).

**TABLE 7.** Resumption of work

*Work resumption*	*RBL*	*Hemorrhoidectomy*	*RR (95% CI*)
After 1 wk, n (%)	23 (85)^a^	10 (44)^a^	2.00 (1.76–2.25)
After number of days, median (IQR)	1 (0–13)	9 (5–15.5)	*p* = 0.021^b^

IQR = interquartile range; RBL = rubber band ligation; RR = relative risk ratio.

a Denominator is the number of patients returning the questionnaire.

b Mann-Whitney-*U* test.

### Patient-Reported Outcome Measures

Over 24 months, PROMs showed significant improvements favoring the hemorrhoidectomy group in HSS, PROM-HISS, and EQ-5D-5L scores, whereas no differences were found in ProctoPROM or Vaizey scores (Table 8). Both groups showed overall improvement in all PROM scores compared to preprocedure levels (see Supplemental Tables 5–10 at http://links.lww.com/DCR/C470).

**TABLE 8.** Patient-reported outcome measures during a 24-mo follow-up

*Scores during 24-mo period*	*RBL*	*Hemorrhoidectomy* *difference (95% CI*)	*p*
HSS score^a^	8.795	–1.122 (–1.938 to –0.306)	0.008
ProctoPROM score^a^	24.409	–1.248 (–4.072 to 1.576)	0.381
PROM-HISS^a^	2.959	–0.247 (–0.426 to –0.068)	0.008
Vaizey score^a^	4.764	–0.300 (–1.661 to 1.051)	0.662
EQ-5D-5L score^b^	0.859	+0.033 (0.005 to 0.061)	0.023

Linear mixed model, corrected for center.

EQ-5D-5L = European Quality of Life 5 Dimensions 5 Level; HSS = hemorrhoidal symptom severity; proctoPROM = proctology patient-reported outcome measure; PROM = patient-reported outcome measure; PROM-HISS = PROM–Hemorrhoidal Impact and Satisfaction Score; RBL = rubber band ligation.

a For these scores a lower score is better.

b For EQ-5D-5L higher score is better.

## DISCUSSION

The HollAND trial was the first RCT to compare RBL and hemorrhoidectomy for grade III hemorrhoids, assessing both effectiveness and patient perspectives. Results showed that the RBL group did not meet the noninferiority criteria for HR-QoL compared to the hemorrhoidectomy group after 12 months. This may be attributed to the sample size not being reached, potentially limiting analysis power. Although hemorrhoidectomy had higher mean QALY scores, the design limits any conclusion of its superiority over RBL. Nevertheless, the QALY differences suggest hemorrhoidectomy may offer better symptom control and improve QoL more effectively.

At baseline, both groups had similar HR-QoL scores. Although the RBL group reported higher scores shortly after treatment, likely due to greater impact of hemorrhoidectomy on daily life and postoperative pain, hemorrhoidectomy showed greater improvement from 6 weeks onward. By 24 months, both treatments had a positive effect on patients, with no significant difference in long-term HR-QoL.

Recurrence rates at 12 months were notably higher after RBL than after hemorrhoidectomy (48.8% vs 6.1%), with the RBL group showing worse outcomes in self-reported recurrence rates (24% vs 0%) and the need for further treatment (24% vs 3%). Consequently, hemorrhoidectomy appears more effective clinically. However, within our study design constraints, we can only assert that RBL is not noninferior.

We used the dichotomous self-reporting method developed by Shanmugam et al.^6–8^ Because posttreatment physical examinations often fail to fully capture patient symptoms, self-reported outcomes add to a more comprehensive measure of treatment success.^24^ In the present study, 54% of RBL patients required further treatment, with 28% undergoing a second banding as permitted by protocol, reflecting the common practice of repeated RBL for grade III hemorrhoids. Among these patients, 42% reported recurrence at 12 months, highlighting that solely tracking reoperations is not sufficient. This is consistent with the Hubble trial, which reported a 49% recurrence rate for grade II–III hemorrhoids after 1 year. In addition, although self-reported recurrence might be influenced by the lack of blinding because patients who are aware of their less invasive treatment might be more inclined to report symptoms, our findings highlight the importance of including self-reported data alongside reoperations for a comprehensive long-time follow-up. The post hoc analysis in the Hubble trial further demonstrated that reclassification of repeated banding in the RBL group significantly reduced recurrence rates from 49% to 37.5%, underscoring the need to compare repeated RBL with hemorrhoidectomy.^8^

Current Dutch guidelines for grade III hemorrhoids include shared decision-making, presenting hemorrhoidectomy or initial RBL as options, and recommending surgery if symptoms persist after 4 RBL sessions.^25^ In our study, only 1 patient exceeded this threshold, opting out of surgery.

In the hemorrhoidectomy group, 1 patient required a second Ligasure procedure, which successfully resolved symptoms. No differences in outcomes were observed between open and closed excisional techniques. Self-reported recurrence rates in this study were lower than those reported in the eTHoS trial (0% vs 14% at 12 months and 6.7% vs 25% at 24 months), likely due to our focus on grade III hemorrhoids.^6^ Conversely, recurrence rates were higher than those reported in our previous meta-analysis (31% for RBL vs 6% for hemorrhoidectomy).^11^ However, the studies included in the meta-analysis were of poor quality, particularly in their definitions of recurrence and follow-up periods.

The skewed treatment allocation (47 vs 40) resulted from stratification by the center with permuted blocks of random sizes, but baseline characteristics remained comparable. Notably, 42% of patients met the Rome IV criteria for functional constipation, emphasizing the importance of addressing underlying bowel issues when planning hemorrhoid treatment. The shorter waiting time of the RBL for intervention may provide an advantage in quickly managing severe symptoms. However, the median waiting time for surgery in our study (50 days; IQR, 27–88) was still shorter than reported in other studies.^6,8^

Both groups experienced minimal complications, indicating that RBL and hemorrhoidectomy are safe treatment options for grade III hemorrhoids. Our findings align with those of previous trials, showing a slight reduction in mean Vaizey scores after both interventions, probably attributed to prolapse correction.^6,8^

Initial postoperative pain scores indicate that RBL provides better pain control, reduced analgesic use, and faster recovery compared to hemorrhoidectomy, with patients returning to work much sooner (1 vs 9 days). For those prioritizing a quicker return to normal activities, RBL may be the preferred option, though this must be weighed against its higher recurrence rates.

Although the hemorrhoidectomy group consistently exhibited better symptom scores during follow-up, the differences were not clinically significant. The average PROM-HISS difference was 0.247, below the minimally relevant difference threshold of 0.3.^26^ However, long-term treatment effects favor hemorrhoidectomy, with differences slightly exceeding the threshold. HSS scores were 1.1 units lower for the hemorrhoidectomy group, but the minimally relevant difference is not clearly defined.^18^ Similarly, a 2-point difference at long-term follow-up was observed, but its clinical relevance remains uncertain. ProctoPROM analysis revealed clinically relevant effects for both treatments, with small differences between groups increasing at 12 to 24 months follow-up.^21^ Overall, although hemorrhoidectomy may offer some long-term benefits, the clinical significance of these differences is unclear.

The main limitation of the study was its premature termination due to funding constraints, leading to a smaller sample size and reduced statistical power. This was largely a result of the impact of the COVID-19 pandemic on benign proctologic care in the Netherlands, causing a shortage of eligible patients. In addition, patient preference delayed enrollment, with 72% of 303 eligible patients excluded, undermining validity and generalizability of the trial.^27^ Many patients were unwilling to undergo surgery as primary treatment, whereas others preferred surgery due to prior RBL failure. Furthermore, the possibility of immediate RBL treatment during initial consultations in Dutch hospitals contributed to the high exclusion rate.

These challenges suggest RCTs may be unsuitable for comparing invasive versus minimally invasive treatments. Considering patient preferences in study designs is crucial because these preferences can lead to reduced recruitment rates and randomization bias, thus compromising the validity of the study.^28^ Future studies should explore alternative trial designs, such as partially randomized patient preference trials and other innovative approaches beyond traditional RCTs to overcome recruitment challenges and improve study validity.^29,30^

Despite its limitations, our study achieved a high questionnaire response rate during 2 years (76%–91%), with 87% completing the 12-month survey. This robust data collection provided reliable long-term treatment evaluations that could aid treatment decisions. Moreover, this study is among the first to prioritize PROMs, reflecting an important step toward integrating patient perspectives into treatment evaluations. The high completion rates and clear treatment preferences expressed by patients highlight the importance of personalized counseling and tailored treatment strategies for enhancing outcomes and satisfaction.

## CONCLUSIONS

Although the present study did not achieve the planned sample size, it provides several key insights. Patients with grade III hemorrhoids may benefit from knowing that although hemorrhoidectomy may involve a longer waiting period, it generally offers a more durable solution with a lower risk of recurrence. However, this procedure is associated with significantly more pain in the initial week, higher analgesic use, and a longer recovery period. Both treatments are deemed safe, but hemorrhoidectomy tends to result in a better QoL and symptom scores, according to patient reports, although these differences were not statistically significant.

## ACKNOWLEDGMENTS

The HollAND Study Group: The principal investigators I.J.M. Han-Geurts, surgeon at the Proctos Kliniek and W.A. Bemelman, surgeon at the Amsterdam University Medical Center (UMC); coordinating investigators: J.Y. van Oostendorp and L. Dekker; and study collaborators: C.I.M. Baeten, surgeon at Groene Hart Ziekenhuis; S.O. Breukink, surgeon at Maastricht UMC; S.M.M. de Castro, surgeon at Onze Lieve Vrouwe Gasthuis; V. Meij, surgeon at Central Military Hospital Utrecht; O. van Ruler, surgeon at Ijsselland ziekenhuis; A.H.W. Schiphorst, surgeon at Diakonessenhuis; R. Schouten, surgeon at Flevoziekenhuis; R. Veldkamp, surgeon at Proctos Kliniek; and S. van Dieren, epidemiologist/statistician at Department of Surgery Amsterdam UMC.

The authors thank M.C. van der Weide for her valuable assistance with the methodology of the trial. They also thank the trial support department at Leading the Change for their help in executing the trial. Finally, they acknowledge the entire team at Proctos Kliniek for their dedicated support throughout the study.

## Supplementary Material
