# Supplementary material for: Comparison of Rubber Band Ligation and Hemorrhoidectomy in Patients With Symptomatic Hemorrhoids Grade III: A Multicenter, Open-Label, Randomized Controlled Noninferiority Trial
Source: Dis Colon Rectum. 2025 Feb 14;68(5):572–83. doi: 10.1097/DCR.0000000000003679 (PMC11999089; doi:10.1097/DCR.0000000000003679)
Supplement: Supplementary file 1 [file dcr-68-0572-s002.pdf]

Appendix

Supplementary Table 1 – Changes made to the protocol after commencement of the trial

Changes to the protocol	Amendment number	Progress report	Date approval	Approved by
Protocol version 7.0: The screening and recruitment process was altered by introducing a telephone recruitment appointment with the study team before the clinical assessment at the outpatient clinic, aiming to maximize recruitment rates and improve informed decision-making.	3	1	13 December 2019	Medical Ethical Committee Amsterdam UMC
Protocol version 8.0: the criterion 'use of oral anticoagulants' was removed from the exclusion criteria list. This adjustment was prompted by the number of excluded patients and the presence of a well-established national perioperative anticoagulation bridging protocol.	8	1	20 August 2020	Medical Ethical Committee Amsterdam UMC
Protocol version 9.0: During the COVID pandemic, patients needing extra time for decision-making could provide consent via phone or email with a photocopy of their signed form. A hardcopy was then obtained at their first hospital appointment.	12	2 (6 October 2021)	15 March 2021	Medical Ethical Committee Amsterdam UMC
Protocol version 10.0: Notification of the premature termination of the study, effective September 22, 2022, due to the discontinuation of funding by the grant provider, ZonMw.	13	3 (final) (20 December 2022)	1 March 2023	-
Protocol version 11.0: The analysis of the primary endpoint, health-related quality of life, was shifted from 24 months to 12 months post-treatment due to the premature termination of the trial resulting from slow inclusion rates. The slow inclusion rate was attributed primarily to the COVID-19 pandemic, which necessitated a significant reduction in proctologic care, as well as patient and physician preferences for specific treatments.	14	-	7 August 2023	Medical Ethical Committee Amsterdam UMC

Supplementary Table 2 – Trial scheme with the time planning of various questionnaires

Study parts	*baseline*	*Study procedures*	*day 1*	*Week 1*	*Week 6*	*6 months*	*12 months*	*24 months*
Clinical evaluation	○			○	○	○	○	○
Treatment details		○						
Patient preference	○							
EQ-5D-5L	○		●	●	●	●	●	●
proctoPROM	○			●	●	●	●	●
PROM-HISS	○			●	●	●	●	●
Rome IV criteria	○				●			
HSS	○				●	●	●	●
Vaizey	○				●	●	●	●
Pain VAS			●	●	●			
Analgesics use			●	●	●			
Return to work			●	●	●			
Self-reported recurrence					●	●	●	●
Further treatment					●	●	●	●
iMCQ					●	●	●	●
iPCQ					●	●	●	●

Supplementary Table 3 – Further treatment for hemorrhoids

Further treatment for hemorrhoids since the index procedure	RBL n=46	Hemorrhoidectomy n=33
**Total, n (%)**	**25 (54%)**	**1 (3%)**
1 additional RBL procedure (**= allowed by protocol**)	13 (28%)	-
2 or more additional RBL procedures	3 (6.5%)	-
1 RBL + hemorrhoidectomy	3 (6.5%)	-
Hemorrhoidectomy	6 (13%)	1 (3%)

RBL = rubber band ligation

Supplementary Table 4 – Self-reported recurrence via questionnaires

Self-reported recurrence, n (%)	RBL	Hemorrhoidectomy	ARD (95% CI)	p-value*
6 weeks	6/23 (26.1%)±	1/22 (4.5%)±	0.22 (0.02 – 0.42)	0.096
6 months	3/38 (7.9%)±	1/33 (3%)±	0.05 (-0.05 – 0.15)	0.618
12 months	9/38 (23.7%)±	0/32 (0%)±	0.24 (0.10 – 0.37)	0.003
24 months	11/37 (29.7%)±	2/30 (6.7%)±	0.23 (0.06 – 0.40)	0.028

± Denominator is the number of patients returning the questionnaire. * Fisher's Exact Test, 2-sided p-values. ARD = absolute risk difference, CI = confidence interval.

Supplementary Table 5 – Vaizey incontinence scores

Vaizey score	RBL	Hemorrhoidectomy	Difference (95% CI)	p-value*
**Baseline**				
N	40	32		
Mean (SD)	4.1 (3.8)	4.5 (4.2)	-0.4 (-2.3 to 1.5)	N/a
Median (IQR)	4 (0.25-6)	4 (0.25-8)		
**Week 6**				
N	40	32		
Mean (SD)	4.2 (3.8)	4.0 (3.9)	0.2 (-1.6 to 2.0)	0.701
Median (IQR)	4 (1-6)	3.5 (0-7.75)		
**Month 6**				
N	38	33		
Mean (SD)	3.3 (3.3)	2.6 (3.3)	0.7 (-0.9 to 2.3)	0.268
Median (IQR)	3 (0-6)	1 (0-4)		
**Month 12**				
N	38	32		
Mean (SD)	3.8 (3.5)	2.7 (3.7)	1.1 (-0.6 to 2.9)	0.108
Median (IQR)	4 (0-6)	1 (0-4)		
**Month 24**				
N	37	30		
Mean (SD)	3.2 (3.4)	3.4 (4.2)	-0.1 (-2.1 to 1.9)	0.774
Median (IQR)	2 (1-5)	2 (0-5.25)		

* Mann Whitney-U Test, 2-sided p-values. N/a = not applicable as it is a randomized study.

Supplementary Table 6 – EQ-5D-5L Index scores

EQ-5D-5L index	RBL	Hemorrhoidectomy	Difference (95% CI)	p-value*
**Baseline**				
N	40	32		
Mean (SD)	0.848 (0.137)	0.812 (0.150)	0.036 (-0.03 to 0.10)	N/a
Median (IQR)	0.887 (0.78-0.98)	0.839 (0.72-0.89)		
**Day 1**				
N	37	29		
Mean (SD)	0.796 (0.195)	0.604 (0.271)	0.192 (0.08 to 0.31)	<0.001
Median (IQR)	0.822 (0.75-0.90)	0.727 (0.49-0.79)		
**Week 1**				
N	39	30		
Mean (SD)	0.870 (0.139)	0.657 (0.219)	0.213 (0.13 to 0.30)	<0.001
Median (IQR)	0.998 (0.82-1.0)	0.717 (0.57-0.81)		
**Week 6**				
N	40	32		
Mean (SD)	0.899 (0.102)	0.939 (0.106)	-0.04 (-0.09 to 0.01)	0.027#
Median (IQR)	0.887 (0.85-1.0)	1.0 (0.88-1.0)		
**Month 6**				
N	38	33		
Mean (SD)	0.914 (0.117)	0.971 (0.06)	-0.06 (-0.10 to -0.01)	0.008#
Median (IQR)	0.957 (0.89-1.0)	1.0 (1.0-1.0)		
**Month 12**				
N	38	32		
Mean (SD)	0.919 (0.08)	0.972 (0.06)	-0.05 (-0.09 to -0.02)	0.003#
Median (IQR)	0.887 (0.85-1.0)	1.0 (1.0-1.0)		
**Month 24**				
N	37	30		
Mean (SD)	0.910 (0.08)	0.940 (0.09)	-0.03 (-0.07 to 0.02)	0.112
Median (IQR)	0.887 (0.85-1.0)	1.0 (0.89-1.0)		

* Mann Whitney-U Test, 2-sided p-values. N/a = not applicable as it is a randomized study.

# Effect changes significantly to the other group.

Supplementary Table 7 – EQ-VAS score

EQ-VAS score	Total	RBL	Hemorrhoidectomy	p-value*
**Baseline**				
N	72	40	32	
Mean (SD)	81 (13.5)	80 (15)	82 (11)	
Median (IQR)	82.5 (75-90)	80 (75-90)	84 (71-90)	N/a
**Day 1**				
N	66	37	29	
Mean (SD)	74 (17.9)	76 (19)	71 (16)	
Median (IQR)	75.5 (61-86)	80 (61-92)	75 (63-81)	0.181
**Week 1**				
N	69	39	30	
Mean (SD)	74 (16.4)	78 (17)	70 (14)	
Median (IQR)	75 (63.5-90)	81 (65-90)	68.5 (60-80)	0.032
**Week 6**				
N	72	40	32	
Mean (SD)	83 (14.1)	81 (15)	85 (12)	
Median (IQR)	82.5 (76-91)	83 (73-90)	82.5 (80-95)	0.276
**Month 6**				
N	71	38	33	
Mean (SD)	83 (15.8)	81 (19)	86 (10)	
Median (IQR)	85 (78-91)	84 (75-91)	90 (80-93)	0.339
**Month 12**				
N	70	38	32	
Mean (SD)	82 (11.6)	82 (13)	83 (10)	
Median (IQR)	82 (75-90)	83 (74-91.5)	82 (75-90)	0.851
**Month 24**				
N	67	37	30	
Mean (SD)	81.5 (11.9)	82 (14)	80 (9)	
Median (IQR)	83.5 (71.5-91)	86.5 (71-92)	80.5 (73-86)	0.354

* Mann Whitney-U Test, 2-sided p-values. N/a = not applicable as it is a randomized study.

Supplementary Table 8 – PROM-HISS score

PROM-HISS	RBL	Hemorrhoidectomy	Difference (95% CI)	p-value*
**Baseline**				
N	40	32		
Mean (SD)	2.6 (0.7)	2.5 (0.7)	0.11 (-0.21 to 0.44)	N/a
Median (IQR)	2.6 (2-3)	2.4 (1.85-3.0)		
**Week 1**				
N	39	30		
Mean (SD)	2.2 (0.8)	2.8 (0.7)	-0.69 (-1.06 to -0.32)	<0.001
Median (IQR)	2 (1.4-2.8)	3 (2.2-3.5)		
**Week 6**				
N	40	32		
Mean (SD)	2.1 (0.8)	1.5 (0.6)	0.58 (0.23 to 0.92)	<0.001#
Median (IQR)	2 (1.4-2.4)	1.2 (1-1.75)		
**Month 6**				
N	38	33		
Mean (SD)	1.7 (0.7)	1.4 (0.4)	0.34 (0.07 to 0.61)	0.017#
Median (IQR)	1.5 (1.2-2)	1.2 (1-1.6)		
**Month 12**				
N	38	32		
Mean (SD)	1.7 (0.6)	1.4 (0.4)	0.35 (0.10 to 0.60)	0.005#
Median (IQR)	1.6 (1.2-2.1)	1.2 (1-1.6)		
**Month 24**				
N	37	30		
Mean (SD)	1.8 (0.5)	1.5 (0.5)	0.33 (0.06 to 0.59)	0.007#
Median (IQR)	1.8 (1.4-2.2)	1.4 (1-1.6)		

* Mann Whitney-U Test, 2-sided p-values. N/a = not applicable as it is a randomized study.

# Effect changes significantly to the other group.

Supplementary Table 9 – HSS score

HSS score	RBL	Hemorrhoidectomy	Difference (95% CI)	p-value*
**Baseline**				
N	40	32		
Mean (SD)	7.4 (2.7)	7.9 (3.3)	-0.5 (-1.9 to 0.9)	N/a
Median (IQR)	7 (5-9)	8 (5.25-11)		
**Week 6**				
N	40	32		
Mean (SD)	5.3 (3.7)	1.7 (2.4)	3.6 (2.1 to 5.1)	<0.001
Median (IQR)	5 (2-7)	1 (0-2)		
**Month 6**				
N	38	33		
Mean (SD)	3.5 (3.2)	1.5 (2.2)	2.0 (0.7 to 3.3)	0.002
Median (IQR)	3 (1-6)	1 (0-2)		
**Month 12**				
N	38	32		
Mean (SD)	3.7 (3.0)	1.5 (1.9)	2.1 (0.9 to 3.4)	<0.001
Median (IQR)	3 (1-6)	1 (0-2)		
**Month 24**				
N	37	30		
Mean (SD)	3.8 (2.6)	1.7 (2.0)	2.0 (0.8 to 3.3)	<0.001
Median (IQR)	3 (2-5.25)	1.5 (0-3)		

* Mann Whitney-U Test, 2-sided p-values. N/a = not applicable as it is a randomized study.

Supplementary Table 10 – ProctoPROM score

ProctoPROM score	RBL	Hemorrhoidectomy	Difference (95% CI)	p-value*
**Baseline**				
N	40	32		
Mean (SD)	19.9 (10.9)	21.9 (10.8)	-2.0 (-7.1 to 3.1)	N/a
Median (IQR)	18 (12-28.6)	22 (14.25-30)		
**Week 1**				
N	39	30		
Mean (SD)	14.5 (12.5)	28.7 (10.7)	-14.2 (-19.9 to -8.5)	<0.001#
Median (IQR)	10 (4-22.5)	32.25 (20.7-37.3)		
**Week 6**				
N	40	32		
Mean (SD)	13 (11.4)	5.1 (10.3)	7.9 (2.7 to 13.0)	<0.001
Median (IQR)	10 (10-22.5)	1 (0-4)		
**Month 6**				
N	38	33		
Mean (SD)	9 (11.1)	4.0 (6.5)	5.0 (0.7 to 9.4)	0.016
Median (IQR)	4.5 (1.2-12.8)	1 (0-6)		
**Month 12**				
N	38	32		
Mean (SD)	9 (11.1)	3.3 (5.0)	5.3 (1.5 to 9.0)	0.002
Median (IQR)	5 (2-12)	2 (0-5)		
**Month 24**				
N	37	30		
Mean (SD)	9.1 (9.0)	5.1 (7.0)	4.0 (-0.3 to 8.3)	0.032
Median (IQR)	8 (2.5-12)	2.5 (0-7)		

* Mann Whitney-U Test, 2-sided p-values. N/a = not applicable as it is a randomized study.

Effect changes significantly to the other group
